# Genetic engineering a large animal model of human hypophosphatasia in sheep

**DOI:** 10.1038/s41598-018-35079-y

**Published:** 2018-11-16

**Authors:** Diarra K. Williams, Carlos Pinzón, Shannon Huggins, Jane H. Pryor, Alyssa Falck, Forrest Herman, James Oldeschulte, Michael B. Chavez, Brian L. Foster, Sarah H. White, Mark E. Westhusin, Larry J. Suva, Charles R. Long, Dana Gaddy

**Affiliations:** 1 Department of Veterinary Physiology and Pharmacology, College of Veterinary Medicine and Biomedical Sciences, Texas A&M University, College Station, TX 77843 USA; 2 Department of Veterinary Integrative Biosciences, College of Veterinary Medicine and Biomedical Sciences, Texas A&M University, College Station, TX 77843 USA; 3 Division of Biosciences, College of Dentistry, The Ohio State University, Columbus, OH 43210 USA; 4 Department of Animal Science, College of Agriculture and Life Sciences, Texas A&M University, College Station, TX 77843 USA

## Abstract

The availability of tools to accurately replicate the clinical phenotype of rare human diseases is a key step toward improved understanding of disease progression and the development of more effective therapeutics. We successfully generated the first large animal model of a rare human bone disease, hypophosphatasia (HPP) using CRISPR/Cas9 to introduce a single point mutation in the tissue nonspecific alkaline phosphatase (TNSALP) gene (*ALPL*) (1077 C > G) in sheep. HPP is a rare inherited disorder of mineral metabolism that affects bone and tooth development, and is associated with muscle weakness. Compared to wild-type (WT) controls, HPP sheep have reduced serum alkaline phosphatase activity, decreased tail vertebral bone size, and metaphyseal flaring, consistent with the mineralization deficits observed in human HPP patients. Computed tomography revealed short roots and thin dentin in incisors, and reduced mandibular bone in HPP vs. WT sheep, accurately replicating odonto-HPP. Skeletal muscle biopsies revealed aberrant fiber size and disorganized mitochondrial cristae structure in HPP vs. WT sheep. These genetically engineered sheep accurately phenocopy human HPP and provide a novel large animal platform for the longitudinal study of HPP progression, as well as other rare human bone diseases.

## Introduction

Hypophosphatasia (HPP) is a rare genetic disorder with a remarkable range of severity (OMIM 241500, 241510, 146300). The highly variable clinical presentation of the disease is organized into a nosology that includes several major clinical forms (odonto, adult, mild and severe childhood, infantile, perinatal, pseudo, and benign prenatal) spanning less severe phenotypes such as dental complications with no skeletal defects to severe manifestations such as death *in utero*^1^. HPP is biochemically characterized by low serum enzyme activity levels of the tissue nonspecific isoenzyme of alkaline phosphatase (TNSALP)^1^. This biochemical hallmark reflects loss-of-function mutations in the coding *ALPL* gene; currently 365 (mostly missense) HPP-inducing mutations have been identified as of August 10, 2018 (http://www.sesep.uvsq.fr/03_hypo_mutations.php)^1,2^. TNSALP is highly expressed in the skeleton, liver, kidney, and developing teeth^1^, though expression of the membrane-bound homodimeric phosphohydrolase is ubiquitous. In HPP, defective mineralization of bones (resulting in rickets/osteomalacia) and teeth (resulting in dental defects and premature tooth loss) can be explained by accumulation of the TNSALP substrate, inorganic pyrophosphate (PP_i_), a potent inhibitor of mineralization^1^.

Case reports of pediatric patients with HPP have been instrumental in advancing the understanding of disease etiology and knowledge of clinical presentations while documenting its wide-ranging severity^3–7^. However, as with many human diseases, HPP animal models to date have been engineered exclusively in rodents, specifically *Alpl* knockout and transgenic mice^8–12^. Although useful for modeling many important features of HPP, murine models harboring *Alpl* mutations do not faithfully represent the broad spectrum of human HPP clinical presentations. Genetically engineered mice with HPP do not lose their teeth, have no apparent muscle weakness, and no muscle structural or ultrastructural defects have been reported. While a bone-targeted recombinant TNSALP (asfotase alfa) is currently available to successfully treat severely affected pediatric HPP patients^13^, a thorough understanding of the broad phenotypic spectrum and pathophysiological effects of HPP on muscle, tooth and bone physiology is lacking and would prove beneficial for assessing prognosis and treatment for any given patient^7^. Indeed, the exon 10 mutation targeted here is based on an index patient (2 years of age) that was originally identified based on rhizomelia and premature tooth loss, subsequently confirmed as HPP genetically and with severely suppressed age-adjusted serum alkaline phosphatase activity and few  discernable bone abnormalities.

To improve the consistency and utility of animal models in which to study rare bone diseases, we developed the first large animal model of HPP. Unlike mice, *Ovis aries* (sheep) bone organization and osteonal remodeling, as well as tooth development and replacement, are highly analogous to humans^14–20^. Importantly, the sheep TNSALP protein sequence is highly conserved with humans (>90% identity) (Supplemental Fig. 1). For CRISPR/Cas9 introduction of a mutation into the sheep genome, we targeted an HPP-causing *ALPL* mutation (p.Ile359Met; c.1077 C > G)^13^. To date, there have been no reports of genetically engineered point mutations in sheep using CRISPR/Cas9. Crispo *et al*.^21^ generated a myostatin gene (*MSTN*)-null mutation in sheep resulting from frameshift mutations causing premature-stop codons^21^. In addition, others have generated deletions of entire genes such as FGF5^22^, yet reports of the successful generation of missense mutations in large animals are limited to goats^23^ and as yet, none have been generated with the goal of modeling a rare human disease, such as HPP.

Gene editing in large animals represents a promising and clinically significant approach for introducing disease mutations that better model human bone disease than murine models that do not fully recapitulate the disease phenotype. The specific point mutation in sheep *ALPL* reported here accurately replicates the clinical phenotype of human childhood HPP, including the muscle and tooth characteristics that identify this mutation as a driver of odonto-HPP. These observations constitute a major advance in the development of a large (domestic) animal platform for the examination of rare bone diseases such as HPP.

## Results

### Confirmation of *ALPL* exon 10 mutation-specific locus

The human *ALPL* coding exon 10 sequence was compared to the *Ovis aries* (sheep) genome using NCBI and Ensembl databases. A highly conserved region (90% identity) on chromosome 2 of the sheep genome was located via BLASTN analysis. Based on the knowledge that >70% of reported *ALPL* mutations in HPP are missense single point mutations^24^, the human *ALPL* exon 10 translated sequence was aligned to the sheep sequence and revealed a highly conserved amino acid sequence (94% identity, Supplemental Fig. 1). This included conservation of the orthologous isoleucine to methionine (p.Ile359Met; c.1077 C > G) HPP mutation target locus reported to cause both mild HPP in the case of a homozygous mutation^25^ and severe HPP in the context of a compound heterozygous mutation with a p.Asp378Val; c.1133 A > T mutation^13^.

### Single guide RNA(sgRNA) and single stranded oligonucleotide (ssODN) repair templates targeting sheep *ALPL* gene

**Figure 1:**
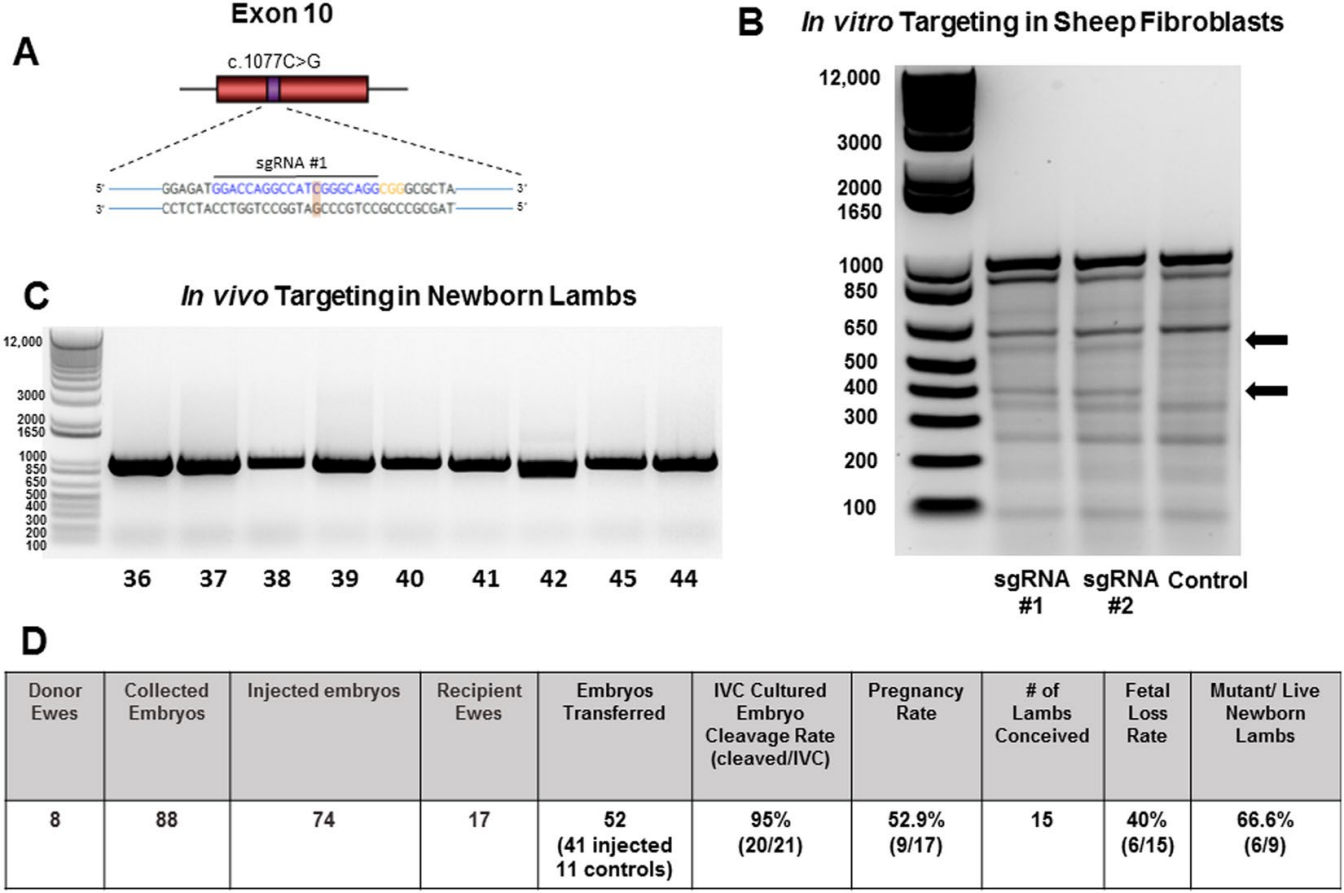
Locus of HPP mutation targeted by sgRNA/Cas9 and targeting efficiency in the *ALPL* sheep gene. (**A**) Schematic of exon 10 (red) with the locus of the HPP c.1077 for the cytosine to guanine (C > G) point mutation (purple). The PAM sequence is in orange where an additional silent point mutation was created to limit Cas9 cleavage. (**B**) Detection of sgRNA/Cas9 targeting efficiency in the *ALPL* gene analyzed with T7E1 assay in sheep fibroblasts. Arrows indicate expected cleaved 600 bp and 400 bp fragment sizes of target regions with sgRNA#1, sgRNA#2, and WT control. (**C**) PCR detection of sgRNA/Cas9 targeting modifications in newborn lambs, individually numbered at the bottom. PCR product (959 bp) flanking target region from DNA extracted from individual newborn lamb umbilicus. (**D**) Summary of genetically-engineered lambs targeting the *ALPL* gene with CRISPR/Cas9. IVC, *in vitro* cultured.

To genetically modify the sheep *ALPL* gene and create a single nucleotide knock-in mutation (c.1077 C > G), two sgRNAs were designed to target exon 10 of the sheep *ALPL* gene with minimal potential off-target genomic effects (Supplemental Table 1). The sequence of sgRNA #1 (GGACCAGGCCATCGGGCAGG, PAM = CGG) and sgRNA #2 (GGCGGGCGCTATGACCTCCG, PAM = TGG) (Fig. 1A) were −5bp and −22bp upstream of the Cas9 cleavage site, respectively. A 91 bp single stranded oligonucleotide repair template with 45 bp homology arms flanking the target site was designed with a silent mutation in the PAM sequence to limit subsequent Cas9 cleavage of the ssODN repair template.

### Targeting of the selected *ALPL* locus in sheep fibroblasts

Functional validation of Cas9 activity and targeting efficiency of sgRNAs were  assessed by transfection of plasmids co-expressing Cas9 and sgRNAs into primary sheep fibroblasts. The T7 endonuclease (T7E1) assay demonstrated efficient targeting of Cas9 by the sgRNAs for genome editing (Fig. 1B). Specific cleavage of the target site was confirmed by Sanger sequencing^26^. Non-specific cleaved fragments were also observed in the T7E1 assay, confirmed by Sanger sequencing to be SNPs in sheep *ALPL* that did not alter amino acid coding sequences (data not shown).

### Genetically-engineered lambs targeting the *ALPL* gene

A total of 88 one or two cell embryos were collected from 8 donor Rambouillet ewes. In sum, 74 *in vitro* genetically manipulated zygotes were generated targeting the *ALPL* exon 10 (c.1077) by microinjection of 10 ng *in vitro* transcribed sgRNA #1, 10 ng Cas9 mRNA, 30 ng Cas9 protein, and either 5 ng or 50 ng of the 91 bp ssODN repair template. Three to four individual embryos (injected n = 41 and/or control n = 11) per recipient were transferred immediately following microinjection into 17 recipient ewes. In addition, 21 microinjected embryos were also *in vitro* cultured (IVC) for analysis. Of the IVC zygotes, a cleavage ratio of 95% was observed 24 hours after culture. To determine gene editing efficiency and subsequent *ALPL* mutation analysis rate, hatched blastocysts (microinjected n = 9; control n = 1) from IVC zygotes were lysed, and whole genome amplified using Qiagen Repli-g Kit per manufacturer’s instructions. The ~1 kb of Exon 10 PCR product flanking the c.1077 C > G target was then generated using forward primer 5′ ATGTTGGGCCCTTTCCCTAA 3′ and reverse primer 5′ TTGGTCCAGGGGTCATGTTG 3′, and Sanger sequencing was performed. Embryo sequencing revealed a mutation rate of 66.6% (4/6), all exhibiting the desired *ALPL* c.1077 C > G point mutation. Nine ewe pregnancies were confirmed by the measurement of Pregnancy-Associated Protein^27^ on day 35 post-transfer, followed by ultrasound for further validation. Fifteen (15) lambs were born 145–152 days after transfer (as expected) with a fetal loss rate of 40% (6/15).

**Figure 2:**
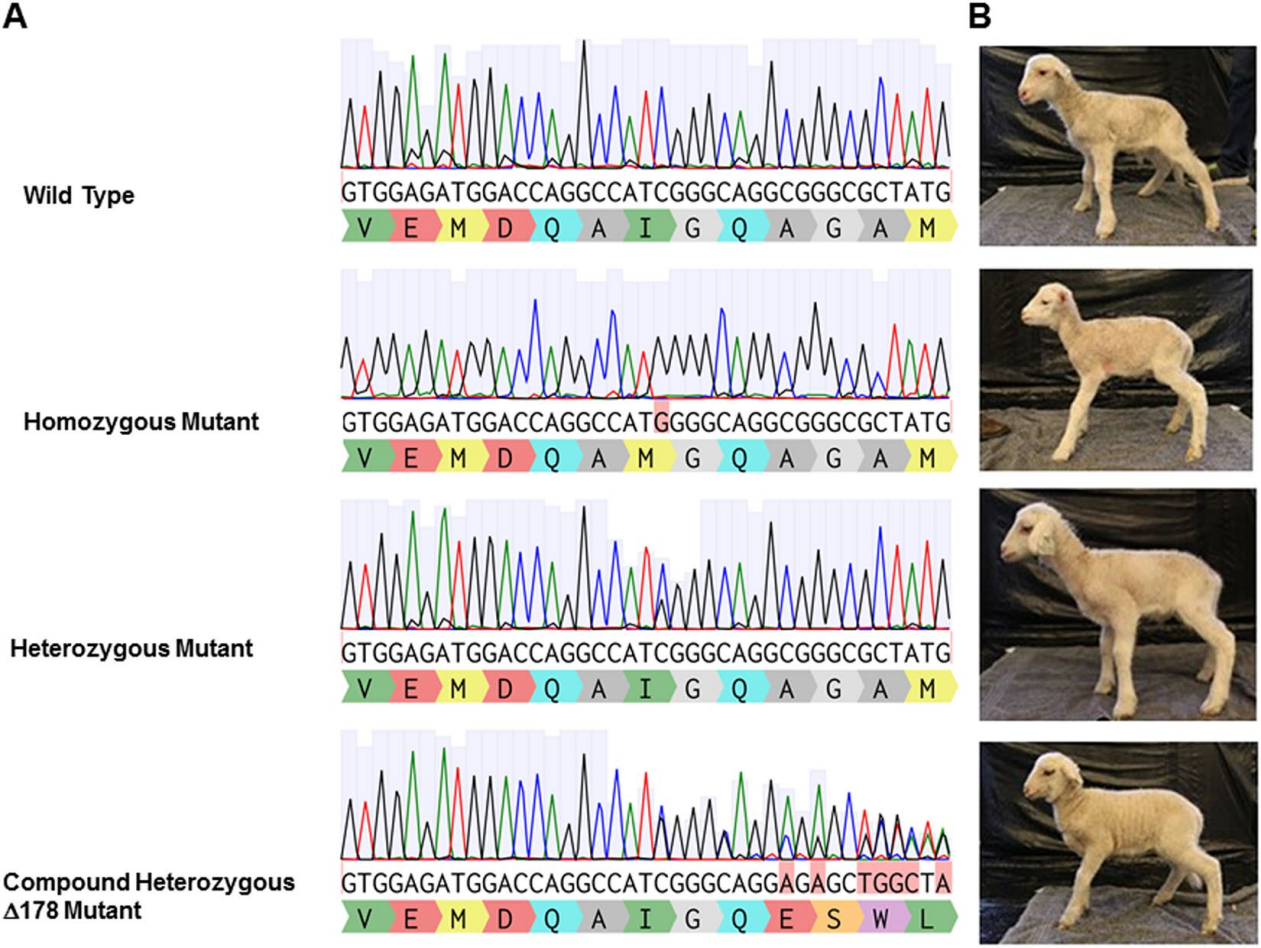
Genotype of *ALPL* c.1077 C > G targeted lambs with CRISPR/Cas9. (**A**) Representative chromatographs from Sanger sequence analysis of newborn lamb umbilicus tissue. (**B**) Photos of ALPL targeted animals from each genotype at 2 weeks of age.

Newborn umbilical cord and whole blood were collected and genomic DNA extracted for analysis. Live newborn lamb DNA was analyzed by PCR (Fig. 1C). Initial examination of PCR products demonstrated an unexpected and aberrant band for lamb 42, with no detectable insertions or deletions for any of the other lambs. Subsequent Sanger sequencing of the 1 kb surrounding the target c.1077 mutation revealed a lamb gene editing rate of 66.6% (6/9) (Fig. 1D). Overall, 3 lambs were wild type (WT), 4 lambs (3 male, 1 female) were confirmed to be heterozygous for *ALPL* exon 10 c.1077 C > G mutation, 1 male lamb homozygous for the mutation, and 1 female compound heterozygote (Fig. 2) with one allele carrying the targeted c.1077 C > G substitution and the other allele carrying a 178 nucleotide deletion (∆178) due to an apparent microhomology just distal to the target site.

### *ALPL* exon 10 c.1077 C > G lambs phenocopy human HPP

**Figure 3:**
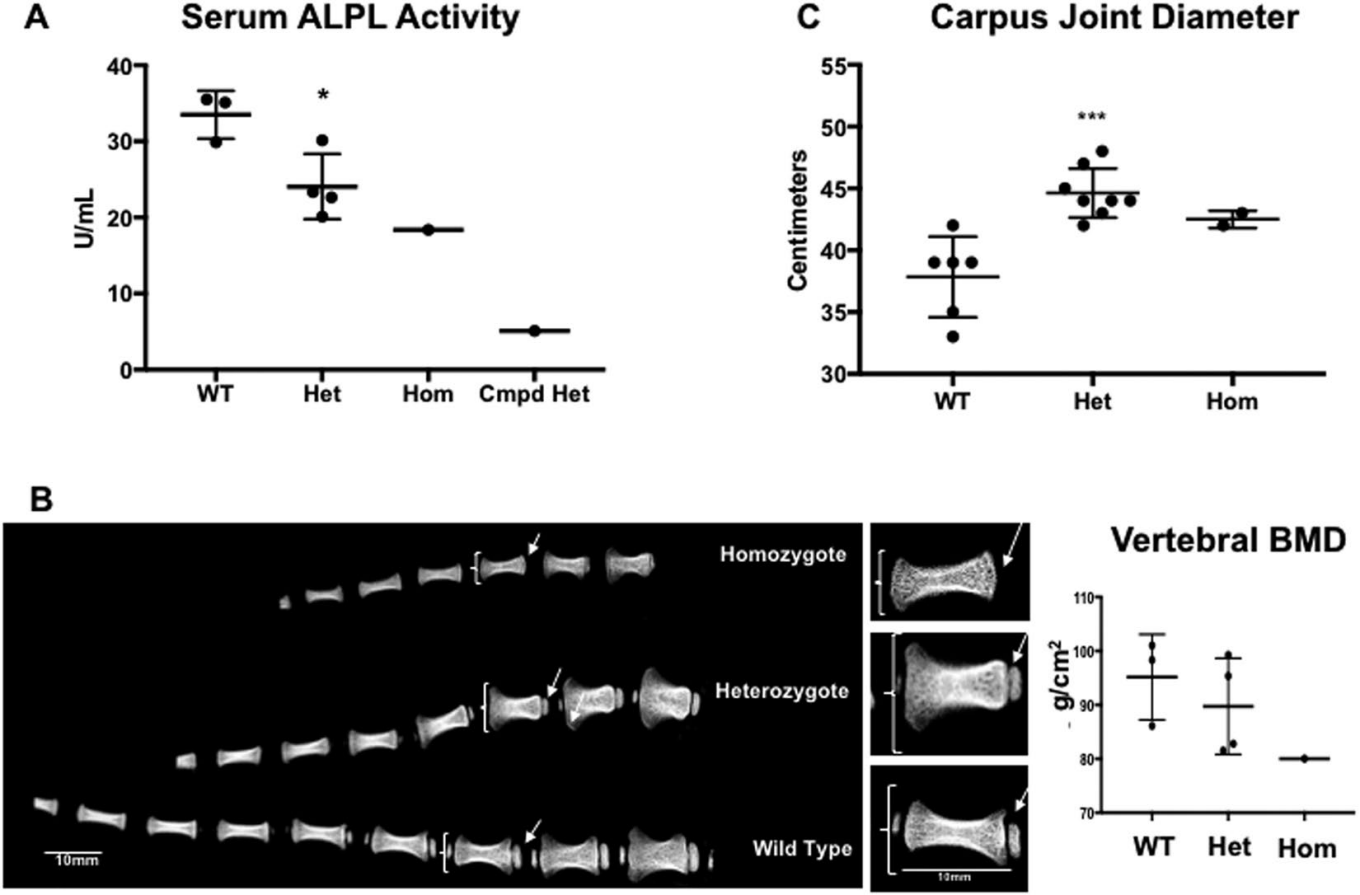
Serum Alkaline Phosphatase activity levels and tail radiographs in *ALPL* c.1077 C > G targeted sheep. (**A**) Serum alkaline phosphatase (ALPL) activity levels in units/mL. Means +/− SEM are shown. *p = 0.0120, by Student’s t-test between wild-type (WT) and heterozygous (Het) groups (n = 3 and 4, respectively). Individual values for the homozygote (Hom) and compound heterozygote (Cmpd Het) are also shown. (**B**) Representative tail radiographs of day 9 lambs obtained by *ex vivo* DXA. Brackets designate distal metaphyses; arrows designate location of secondary ossification centers. Inset magnification of individual tail vertebrae from B shows size and morphology differences in the 3 genotypes. Note different shape, size and extent of mineralization in the individual vertebrae. Vertebral BMD values for the matched distal 4 vertebrae from each group are shown. The small magnitude of difference between groups of small n was NSD by Student’s one-tailed t-test. (**C**) Carpus diameter measurements (2 joints per animal measured at 2 months of age) showing significant increases in heterozygous (Het) animals and increased size in homozygous (Hom), compared with wild type (WT). Means +/− SEM are shown. ***p = 0.0002, t = 4.849, df = 12 by Student’s one-tailed t-test between WT and heterozygous groups (n = 3 and 4, respectively).

Similar to HPP patients with the orthologous c.1077 C > G mutation^25^ and consistent with the biochemical hallmark of human HPP^1^, serum TNSALP (ALPL) activity in c.1077 C > G mutant sheep at 2 months of age was significantly reduced in mutant heterozygotes (from 28–85%) and substantially reduced in the homozygote and compound heterozygote compared to wildtype animals (Fig. 3A). Not surprisingly, given the extent of the ∆178 deletion, the compound heterozygous animal (Fig. 2) had the lowest measured serum TNSALP activity (Fig. 3A). As a result, this animal was not subjected to further detailed phenotypic evaluation based on concerns regarding interpretation being confounded by the complexity of the compound heterozygous genotype.

HPP mutant sheep displayed a variable phenotype expression at birth: Several lambs suffered from respiratory difficulties diagnosed as pneumonia that led to fatalities in 2 mutant animals, similar to respiratory insufficiency in perinatal HPP^28^. Birth weights of the newborn mutant lambs were smaller than their WT counterparts (WT lambs = 5.51 kg ±0.34 SEM; Mutant lambs = 3.85 kg ±0.35 SEM), but grew at similar rates (Supplemental Fig. 2). As in humans, neonatal birth weights are lower with multiple births (twins or triplets) than from singlets. Weekly body weights and rate of weight gain are a standard in the production animal field for size of the offspring and were tracked, showing that the rate of weight gain is similar across all genotypes (Supplemental Fig. 2A). Similarly, pre wean daily weight gain (Supplemental Fig. 2B) and post-wean average daily weight gain (Supplemental Fig. 2C) also showed no significant difference between genotypes. On day 10, DXA analysis of docked tails revealed apparent metaphyseal flaring (brackets), and a virtual lack of secondary ossification centers (arrows) in some mutant vertebrae compared with WT lambs, entirely consistent with the clinical signs of HPP (Fig. 3B), as well as numerically decreased vertebral bone mineral density measurements in mutants (Fig. 3B). Metaphyseal flaring was also readily apparent in carpus joint diameter measurements (Fig. 3C), with the magnitude of difference between genotypes sufficient to demonstrate that heterozygous animals have a significantly larger carpus diameter than their WT counterparts. Similarly, the homozygous mutant had a higher numerical carpus diameter measurement than WT, all indicative of enlarged, flared joints (Fig. 3C) consistent with HPP.

Following DXA analysis, individual formalin fixed lamb vertebral bodies (Fig. 3B insets) were dissected for subsequent microCT and histologic examination (Supplemental Fig. 3). Individual 3D reconstructions (Supplemental Fig. 3A) revealed a similar architecture across genotypes, with porous outer cortex and thick trabeculae in the metaphyseal regions. Note however, the lack of secondary ossification centers in the mutant vertebrae. Next, vertebrae were evaluated histologically (Supplemental Fig. 3B). A similar morphology was observed, with no differences in growth plate architecture or cellular organization.

**Figure 4:**
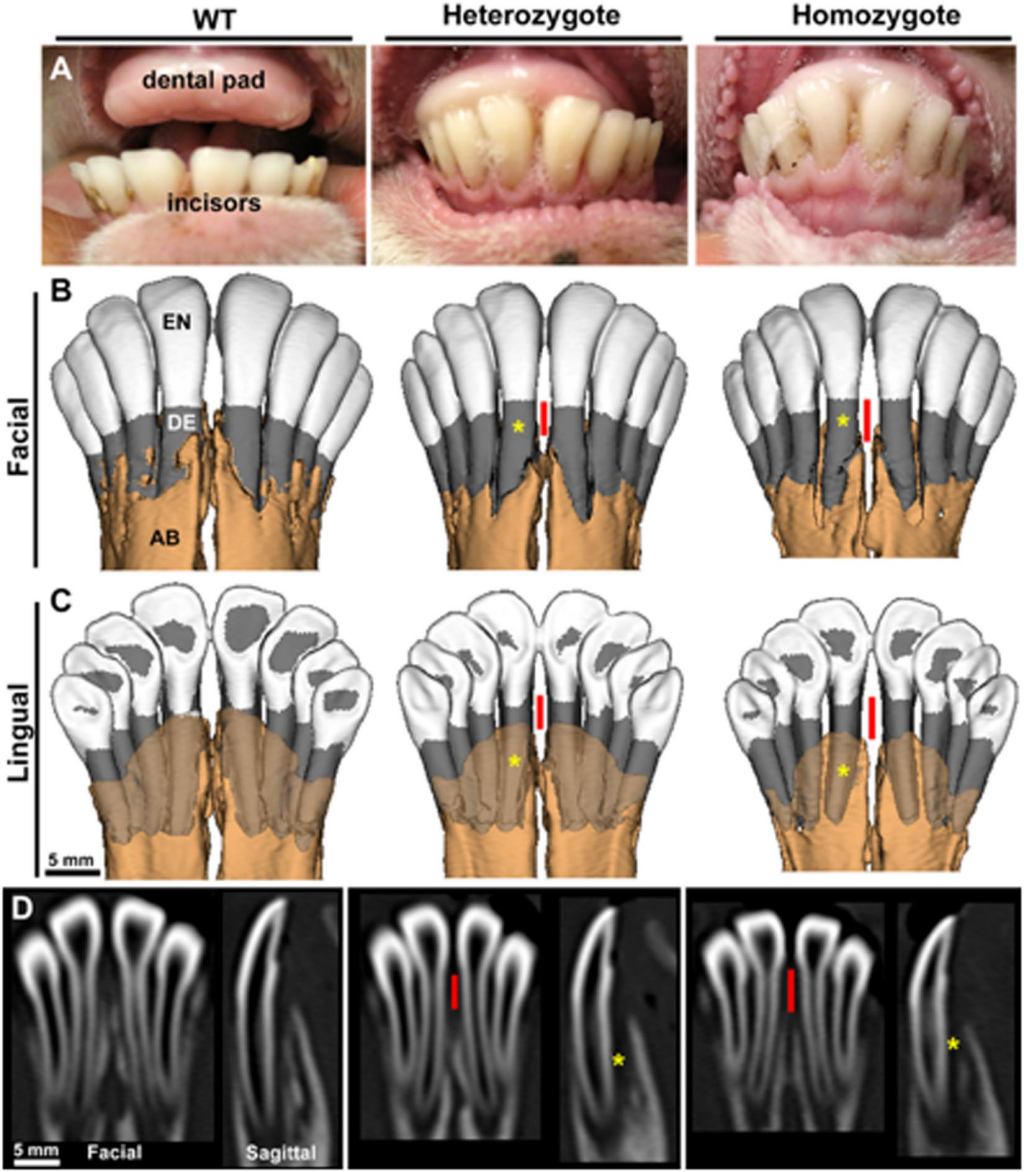
Dental phenotype in *ALPL* c.1077 C > G targeted sheep. (**A**) Oral photographs from wild type (WT), heterozygous, and homozygous lambs at 2 months of age show the maxillary dental pad and mandibular incisors. (**B**) Reconstructions of *in vivo* CT of mandibles at 2 months of age. Enamel (EN) is shown in white, dentin (DE) in gray, and alveolar bone (AB) in light brown. 3D facial view with opaque AB. (**C**) 3D lingual view with translucent AB, and (**D**) 2D facial and sagittal sections through the central incisors, reveal thinner and shorter incisor roots (yellow*), reduced AB, and greater root surface exposure (red lines) in the incisors of heterozygotes and homozygotes compared to WT.

In addition to having evidence of metaphyseal flaring and compromised bone parameters, sheep with *ALPL* mutations also presented significant dentoalveolar alterations. At birth, sheep have a maxillary dental pad and 8 primary incisors in the mandible, which are replaced by secondary incisors by approximately 1 year^29^ (Fig. 4A). *In vivo* computed tomography (CT) analysis at 2 months of age revealed a dental phenotype consistent with defects reported in human HPP^2^. 3D CT facial and lingual views of the mandibular incisors (Fig. 4B,C) and 2D facial and sagittal views (Fig. 4D) reveal thinner and shorter tooth roots (yellow*), reduced alveolar bone levels, and greater root surface exposure (red lines) in the incisors of heterozygotes and homozygotes compared to WT.

**Figure 5:**
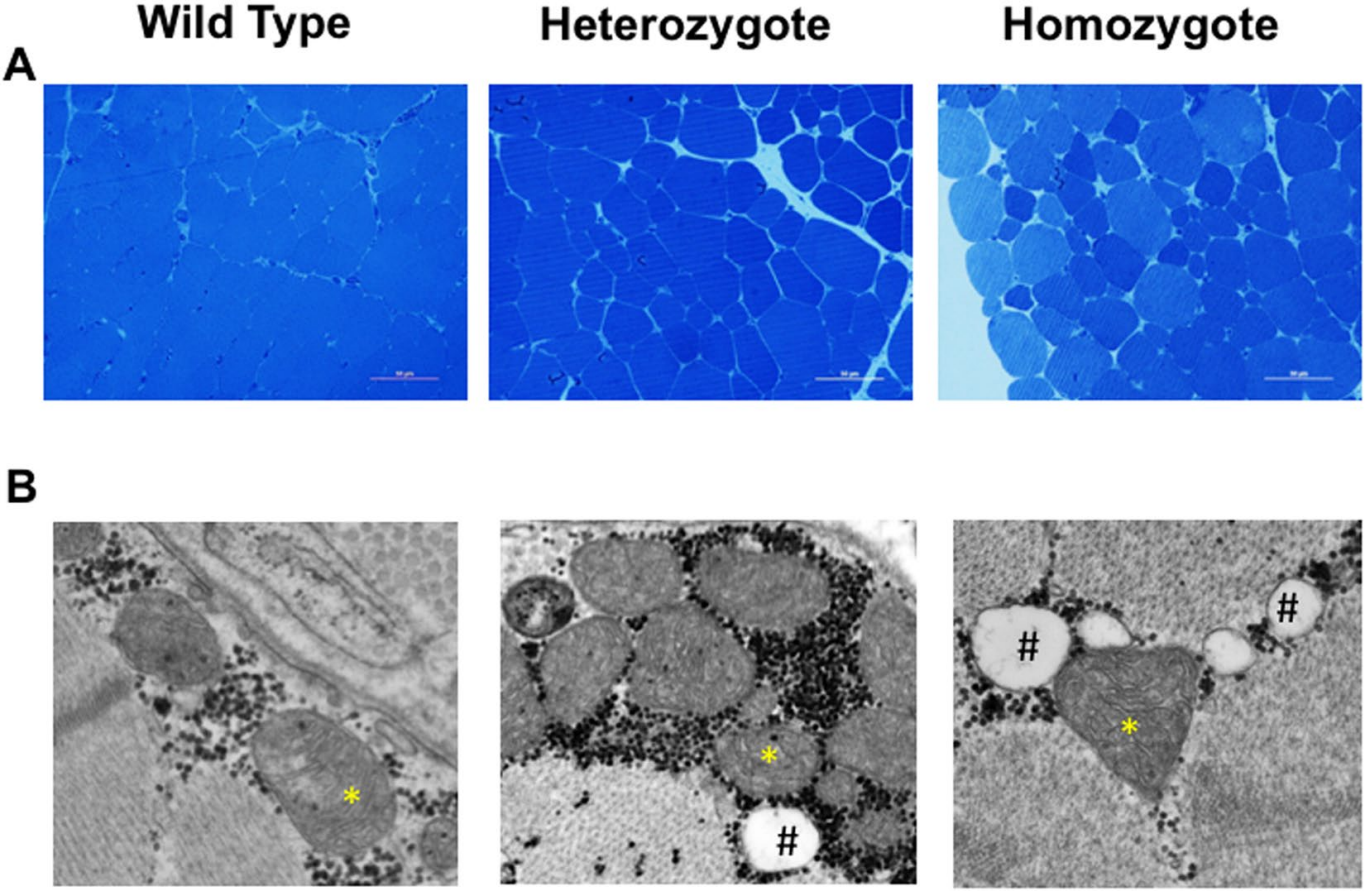
Skeletal muscle histological phenotype in *ALPL* c.1077 C > G targeted sheep. (**A**) Representative light microscopic evaluation (40X magnification, size bar, 50 µm) of Richardson’s stained 500 nm sections of 2 month old gluteus muscle reveal variable sized muscle fibers in mutants compared to homogeneously sized WT fibers. (**B**) Representative electron micrographs (original magnification 44,000X) of HPP mutants compared to WT reveal abnormal mitochondria cristae ultrastructure (*) and higher fat content (#).

Muscle weakness is also characteristic of human HPP, though the underlying pathology is not well understood^1^ and is not observed in murine HPP models^8^. At 2 months of age, mutant sheep showed signs of muscle weakness with a qualitatively altered gait, microscopically aberrant muscle structure as well as changes in mitochondria morphology compared to WT sheep. Muscle histology identified a more variable muscle fiber size (Fig. 5A) and ultrastructurally altered mitochondrial cristae (Fig. 5B). Close examination revealed that mutant mitochondrial cristae were incorrectly folded. The cristae are folds of the inner mitochondrial membrane that increase surface area to facilitate electron transport chain and chemiosmosis, allowing for increased production of ATP. In HPP sheep, these structures are highly disorganized and poorly aligned, likely leading to decreased ATP production^30^.

## Discussion

An ideal animal model of a human disease should satisfy several criteria: (i) the model should closely approximate the condition under investigation; (ii) the model should allow longitudinal study and permit appropriate tests to be performed; and (iii) specific analytical reagents should be available. To date, the use of murine models has been driven almost exclusively by the availability of mouse genetics and their minimal animal costs; the ability to manipulate the rodent genome, the short reproductive cycles, and the generation of a range of genetically identical inbred strains of mice has forced investigators down the murine route^31^. These important advantages have led to the acceptance of often less than accurate murine models as sufficient for research into many human disorders. However, unlike humans, rodents have smaller long bones with thin fragile cortices that lack Haversian remodeling, which is the fundamental process by which larger animals, such as sheep and humans, model and remodel their skeletons throughout life^32–34^. Additionally, mice are monophyodont (one set of teeth), while sheep and humans are diphiodont (two sets of teeth), including primary and secondary sets. In order to more accurately model the associated musculoskeletal and dental phenotypes of human HPP, we utilized domestic sheep as a new animal model.

Collectively, engineering the sheep *ALPL* missense-mutation (c.1077 C > G) model of HPP accurately recapitulated many of the varied clinical manifestations observed in individuals with HPP^34^, satisfying the most stringent criteria for an animal model for the study of human musculoskeletal disease and marking a major advance beyond the existing and largely limited murine models.

The missense mutation engineered into the sheep *ALPL* gene (1077 C > G) was generated using CRISPR/Cas9, resulting in the first large animal model of human HPP. These animals harbored a primary TNSALP deficiency and the associated musculoskeletal and dental phenotype. Gene editing was efficient with a specific point mutation incorporation rate of 66.6%, demonstrating the utility of the sheep genome for precise editing. Children with the identical 1077 C > G mutation have been reported to cause both mild HPP in the case of a homozygous mutation^25^ and severe HPP in the context of a compound heterozygous mutation with an Asp378Val; c.1133 A > T mutation^25,35^. Interestingly, HPP mutant sheep were also born with a variable clinical presentation, a similar mild bone deformity measured as increased carpus diameter, structural muscle deficits and dental defects consistent with the mild form of childhood human HPP (Fig. 3) and odonto-HPP (Fig. 4)^1^. The novel structural and ultrastructural mitochondrial defects observed in muscle biopsies suggest differences in muscle energetics which may provide new insights into the currently unknown mechanism underlying the muscle weakness commonly observed in HPP patients^1,2^. Indeed, the sheep HPP model may provide an opportunity to discern whether muscle weakness is the direct effect of decreased TNSALP activity in muscle, or instead is in response to systemic alterations in inorganic pyrophosphate (PP_i_) due to changes in TNSALP activity in bone. In addition, this model has the potential to uncover mechanisms linking mitochondrial dysfunction to the natural history of the disease. The ovine model also provides opportunities to follow HPP progression longitudinally, allowing repeated bone and muscle biopsies, timed administration of treatment(s), evaluation of primary and secondary tooth development and retention, and long-term assessment of periodontal health and interventions such as orthodontics and dental implants, all of which are the focus of intensive investigation in our laboratory.

The sheep HPP model represents a novel and critical first step in an ongoing effort to study the lifelong etiopathology of HPP. Throughout life, the extracellular accumulation of the primary TNSALP substrate, PP_i_, has been associated with tooth loss, rickets or osteomalacia, and myasthenia in HPP^2^. However, the relative importance of PP_i_ over other known TNSALP substrates (pyridoxal 5′-phosphate and phosphoethanolamine) has not been directly determined and these substrates have been examined only cross-sectionally in groups of affected patients^2^. Indeed, while rodent models have been the mainstay of the bone field and yielded critically important information, sheep are much more physiologically relevant to humans in terms of bone quality, bone remodeling, dental structures and dental development. The sheep HPP model enables repeated sampling of affected animals prospectively with repeated biopsy and detailed molecular interrogation, not currently possible in children with the disorder. This is particularly relevant since HPP-causing mutations in the *ALPL* gene cannot be studied in depth due to the limitations of rodent models that do not develop the extensive tooth or muscle phenotypes^36^.

The recently approved bone-targeted TNSALP enzyme replacement therapy, asfotase alfa, has demonstrated significant improvements in skeletal mineralization, including ribs, resulting in improved respiratory function and survival in life-threatening perinatal and infantile HPP cases^28^. However, the current treatment is only provided to children with severe HPP. In patients with milder forms of HPP, tooth loss, muscle weakness and bone fracture remain important patient care issues. The ovine model described here provides an excellent tool for testing new strategies for bone repair and tooth development in adult and juvenile forms of milder HPP respectively. The impact of the treatment on the skeleton is profound and dramatic, yet little is known regarding the time-course of effects on muscle and other tissues. Importantly, there are no reports of treatment effects (if any) on tooth loss and/or retention, studies that will be possible in this model. Indeed, significant improvements in patient growth, strength, motor function, agility, and quality-of-life have been observed prior to any changes in skeletal mineralization^35^, with details of the underlying mechanism completely unknown. Thus, a thorough understanding of the broad spectrum of the disease in affected patients, as well as the detailed pathophysiological effects of HPP are still lacking. We hypothesize that the HPP sheep described here provide a unique opportunity to address fundamental HPP questions regarding muscle, bone, and tooth development, and will identify new approaches to improve patient care.

## Materials and Methods

### Animals

Animal experiments were performed under an animal use protocol #IACUC 2017-0210 approved by the Texas A&M University Institutional Animal Care and Use Committee (IACUC), and experiments conformed to all guidelines specified in this committee-approved protocol. Anthropomorphic measurements of body temperature (daily) as well as weight (weekly), length (as measured from the sternum (manubrium) to the aitchbone (tuber ischiadicum) and lamb carpus diameter, were performed at 2 weeks and 2 months of age.

### sgRNA design and preparation for microinjection

In order to identify the specific region in the sheep genome for targeting, the NCBI *Ovis aries* V4.0 tissue non-specific alkaline phosphatase (*ALPL*) gene sequence was imported into Benchling (Benchling, San Francisco, CA) and aligned to the human genome using nucleotide BLAST suite (https://blast.ncbi.nlm.nih.gov/). The protein sequence was confirmed using NCBI protein Blast (https://blast.ncbi.nlm.nih.gov/). Following identification and confirmation of the human *ALPL* mutation locus in the sheep *ALPL* gene, primer pairs flanking exon 10 (used to generate a 1 kb PCR product) were designed using Primer3 software (v. 0.4.0)^37,38^ (Forward: 5′ ATGTTGGGCCCTTTCCCTAA 3′, Reverse: 5′ TTGGTCCAGGGGTCATGTTG 3′). Two specific sgRNAs targeting exon 10 were designed using the Benchling web based CRISPR gRNA design tool (Benchling, San Francisco, CA) that would result in minimal off-target effects (Supplemental Table 1). RNA folding was assessed using the WU-CRISPR tool of the Washington University described by Wong, *et al*.^39^ and the Mfold web server for nucleic acid folding and hybridization prediction (http://unafold.rna.albany.edu/?q=mfold/rna-folding-form)^39^. Custom sgRNAs were cloned into the Origene Technologies (Rockville, MD) All-in-One CRIPSR/Cas9 vector (pCas-Guide-EF1a-GFP). For the production of the sgRNA for microinjection into embryos, a T7 *in vitro* transcription of the sgRNA was synthesized^40^ using the MEGAshortscript T7 transcription kit (Thermo Fisher Scientific, Waltham, MA).

### Primary sheep fibroblast cell establishment and culture

Primary sheep fibroblast cultures were established from sheep dermal biopsies. Briefly, skin samples were harvested and placed in Ca^2+^ and Mg^2+^ free Dulbecco’s phosphate buffered saline (DPBS; Life Technologies, Gaithersburg, MD) on ice. The samples were quickly washed in 0.2% (v/v) chlorhexidine gluconate in DBPS, and then washed twice more by sterile DPBS. Each sample was placed in a 10 cm petri dish and cut into small pieces (<5 mm) using sterile instruments. The tissue was then washed through 5 series of 1x DPBS washes in a 12 ml conical tube. Tissue was resuspended in Dulbecco’s Modified Eagle Medium with nutrient mixture F-12 (DMEM/F12; Life Technologies) supplemented with 10% FBS (Atlanta Biologicals, Atlanta, GA), 1x Antibiotics/Mycotics and 1x Gentamycin (Life Technologies). The prepared tissue was then placed in a T25 tissue culture flasks and cultured at 37 °C in a 5% O_2,_ 5% CO_2_ humidified incubator. Cells were passaged when approximately 80% confluent.

### Harvest of *in vivo* matured oocytes and zygotes for production of gene-targeted sheep

Ten (10) mature Rambouillet donor ewes were estrus synchronized by treating with controlled internal drug release (CIDR) devices containing 300 mg progesterone (Zoetis) for 12 days. On the day of CIDR insertion (Day 0), all ewes received 0.25 mg cloprostenol sodium (Merck) intramuscular (i.m.) to induce luteolysis in any corpus luteum (CL) present. Superovulation was induced on days 9–12 with 200 mg of FSH (Bioniche) administered i.m. in eight decreasing doses (40 mg × 2, 30 mg × 2, 20 mg × 2, 10 mg × 2). CIDR devices were removed on day 12, and 0.05 mg GnRH (Merial) was administered i.m. 36 hours later. At 51 hours post CIDR removal, laprascopic artificial insemination was performed using fresh collected ovine semen. Twenty (20) mature cross bred recipient ewes were also synchronized using CIDR devices and 0.25 mg cloprostenol sodium on day 0. CIDR devices were removed on day 12 and 0.05 mg GnRH was administered 36 hours later. Heat was confirmed on the recipient ewes by exposure to epididymectomized rams with marking harnesses.

Zygotes were recovered surgically 24 hours later by mid-ventral laparotomy. For this procedure, sheep were anesthetized with an intravenous (i.v.) injection of a ketamine and xylazine mixture, with anesthesia maintained under isofluorane inhalation. The reproductive tract was exposed and the oviducts were flushed with 25 ml of HEPES-buffered medium into a 50 ml conical tube. Recovered medium was examined under a dissecting microscope and the embryos collected. All recovered *in vivo* zygotes were held at 35 °C in TL-HEPES medium prior to microinjection.

### Zygote microinjection and transfer

Presumptive zygotes were further cleaned with a stripper pipette (125 µm diameter) to remove sperm and any remaining cumulus cells. Cleaned zygotes were placed in Hanks 199 (Gibco-BRL) supplemented with 10% Hyclone FBS (GE) and gentamicin. Zygotes were injected with 10 ng/µl Cas9 mRNA, 30 ng/µl of Cas9 protein, 5 or 50 ng/µl of ssODN repair template, and 5 ng/µl *in vitro* transcribed sgRNA produced as described above. Injections were performed under positive pressure until a slight expansion of the cell membrane was observed. All injected zygotes were placed in 4-well multi-dishes with each well containing 500 μl of culture medium (IVF Bioscience, Falmouth, England) and cultured for 6–7 days at 38.5 °C in a 5% O_2,_ 5% CO_2_ humidified incubator for IVC zygotes. All other zygotes were immediately transferred to recipient ewes under general anesthesia, following a mid-line laparotomy to expose the uterus. A Drummond pipette was used to transfer two or three zygotes into the oviduct ipsilateral to corpus hemmoragicum.

### T7E1 Cleavage Assay

The T7E1 assay was used as described^40^ to determine sgRNA/Cas9 cleavage activity in primary sheep fibroblasts and to confirm Cas9 activity in DNA from newborn lambs. In brief, 3 × 10^5^ sheep fibroblasts were seeded in a 1% gelatin coated 6-well plates and grown until the confluence reached 80%. Cells were transfected with 2.5 μg CRISPR/Cas9 plasmid using Lipofectamine 3000 (Thermo Fisher Scientific) and maintained in culture at 37 °C during 48–72 h according to the manufacturer’s instructions. Transfected cells were harvested and genomic DNA was extracted using the DNA Blood and Tissue Kit (Qiagen, Germantown, MD). The targeted exon 10 region was PCR amplified (~1 kb PCR product) and the amplicon purified using PCR Purification Kit (Qiagen). Next, 200 ng of each purified PCR amplicon was denatured, re-annealed, and digested with T7 endonuclease I (New England Biolabs, MA). Re-annealed heteroduplex fragments were electrophoresed on a 2% TAE agarose (90 v for 3 hours) and enzymatic cleavage visualized. In order to determine gene modifications in newborn lambs, DNA was extracted from skin and blood samples and assessed using the same T7E1 protocol.

### PCR and genomic sequencing

Genomic DNA was extracted from embryos, cells, skin, umbilical cord and blood samples (Qiagen DNeasy Blood and Tissue Kit). PCR amplification was performed using 200 ng of DNA and the Cloneamp HiFi PCR premix (Clontech, Mountain View, CA) according to the following protocol: 95 °C for 5 min followed by 95 °C for 30 sec, 66 °C for 30 sec, 72 °C for 45 sec, and 72 °C for 10 min after 35 cycles. PCR products were gel purified by electrophoresing on a 1% TAE agarose gel (90 v, ~2 h) and gel extracted using QIAquick gel extraction kit (Qiagen). All isolated and purified PCR products were analyzed via Sanger sequencing.

### Sheep Phenotyping

Blood was collected at 2 months of age and serum analyzed for alkaline phosphatase activity at pH 10.4 using *p*-nitrophenol phosphate as colorimetric substrate (Invitrogen). For *in life* CT measurements, all sheep were sedated using midazolam (0.4 mg/kg) and ketamine (1–2 mg/kg) followed by isoflurane general anesthesia administered via face mask for the duration of the CT scans. Animals were placed in sternal recumbence for whole body non-contrast CT imaging (Siemens Biograph mCT, Siemens Healthineers USA). (128 slice scanner, 1.5 mm slice thickness scans with 0.6 mm isotropic voxel reconstructions, 140 kV and 250 mAs). Working reconstructions were performed on the Siemens Syngo workstation (Siemens Healthineers, USA). For dental analyses, CT image stacks (DICOM) were exported as IMA files and reconstructed in AnalyzePro 1.0 (AnalyzeDirect, Overlan Park, KS, USA). Sheep mandibles were oriented to a standard orientation using incisors as landmarks. Enamel was traced above a threshold of 1600 Hounsfield units (HU) and the threshold used for bone and dentin was 400 HU, with some manual correction done as necessary to separate bone and dentin. 3D facial and lingual views were prepared, with the latter rendering alveolar bone translucent to allow a view of incisor roots. 2D facial and sagittal (through right central incisor) views were prepared. Mineralization of the tail vertebrae was analyzed *ex vivo* using a Faxitron Ultrafocus DXA (Tucson, AZ) of tails docked at 10 days of age and formalin fixed. *Ex vivo* microCT analysis of individual dissected formalin fixed sheep caudal vertebrae was performed using Scanco Medical microCT50 (Brüttisellen, Switzerland). The individual vertebrae were scanned as 4 µm^3^ isotropic voxel size using 55 kVp, 114 mA, and 200-ms and subjected to Gaussian filtration and segmentation. Bone was segmented from soft tissue using the same threshold for all animals, 290 mg HA/cm^3^ for trabecular bone. Following microCT imaging, vertebrae were decalcified in 12% EDTA with agitation and the decalcified specimens dehydrated through graded ethanol, cleared and embedded in paraffin, sectioned (5 μm) and stained with hematoxylin and eosin (H&E) as described^41^. Muscle histology and ultrastructure was determined using gluteal muscle biopsies obtained at 2 months of age under anesthesia according to the approved IACUC protocol. Muscle biopsies were then fixed in sodium cacodylate-buffered paraformaldehyde, post-fixed in osmium tetroxide and processed in plastic for light and transmission electron microscopy.

## Electronic supplementary material

Supplementary Information

## References

[1] Whyte MP (2016). Hypophosphatasia - aetiology, nosology, pathogenesis, diagnosis and treatment. Nat Rev Endocrinol.

[2] Whyte MP, Wenkert D, Zhang F (2016). Hypophosphatasia: Natural history study of 101 affected children investigated at one research center. Bone.

[3] Camacho PM (2016). Adult Hypophosphatasia Treated with Teriparatide: Report of 2 Patients and Review of the Literature. Endocr Pract.

[4] Khandwala HM, Mumm S, Whyte MP (2006). Low serum alkaline phosphatase activity and pathologic fracture: case report and brief review of hypophosphatasia diagnosed in adulthood. Endocr Pract.

[5] Whyte MP (1990). Heritable metabolic and dysplastic bone diseases. Endocrinol Metab Clin North Am.

[6] Whyte MP, Essmyer K, Geimer M, Mumm S (2006). Homozygosity for TNSALP mutation 1348c> T (Arg433Cys) causes infantile hypophosphatasia manifesting transient disease correction and variably lethal outcome in a kindred of black ancestry. J Pediatr.

[7] Whyte MP (2017). Hypophosphatasia: Enzyme Replacement Therapy Brings New Opportunities and New Challenges. J Bone Miner Res.

[8] Fedde KN (1999). Alkaline phosphatase knock-out mice recapitulate the metabolic and skeletal defects of infantile hypophosphatasia. J Bone Miner Res.

[9] Foster BL (2015). Periodontal Defects in the A116T Knock-in Murine Model of Odontohypophosphatasia. J Dent Res.

[10] McKee MD (2011). Enzyme replacement therapy prevents dental defects in a model of hypophosphatasia. J Dent Res.

[11] Millan JL, Whyte MP (2016). Alkaline Phosphatase and Hypophosphatasia. Calcif Tissue Int.

[12] Yadav MC (2012). Enzyme replacement prevents enamel defects in hypophosphatasia mice. J Bone Miner Res.

[13] Whyte MP (2012). Enzyme-replacement therapy in life-threatening hypophosphatasia. N Engl J Med.

[14] Delmas PD (1995). The anabolic effect of human PTH (1–34) on bone formation is blunted when bone resorption is inhibited by the bisphosphonate tiludronate–is activated resorption a prerequisite for the *in vivo* effect of PTH on formation in a remodeling system?. Bone.

[15] Chavassieux P (1991). Dose effects on ewe bone remodeling of short-term sodium fluoride administration–a histomorphometric and biochemical study. Bone.

[16] Newman E, Turner AS, Wark JD (1995). The potential of sheep for the study of osteopenia: current status and comparison with other animal models. Bone.

[17] Kreipke TC (2014). Alterations in trabecular bone microarchitecture in the ovine spine and distal femur following ovariectomy. J Biomech.

[18] Les CM (2004). Determinants of ovine compact bone viscoelastic properties: effects of architecture, mineralization, and remodeling. Bone.

[19] Les CM (2005). Long-term ovariectomy decreases ovine compact bone viscoelasticity. J Orthop Res.

[20] Turner AS (2002). The sheep as a model for osteoporosis in humans. Vet J.

[21] Crispo M (2015). Efficient Generation of Myostatin Knock-Out Sheep Using CRISPR/Cas9 Technology and Microinjection into Zygotes. PLoS One.

[22] Hu R (2017). RAPID COMMUNICATION: Generation of FGF5 knockout sheep via the CRISPR/Cas9 system. J Anim Sci.

[23] Niu Y (2018). Efficient generation of goats with defined point mutation (I397V) in GDF9 through CRISPR/Cas9. Reprod Fertil Dev.

[24] Mornet E (2017). Genetics of hypophosphatasia. Arch Pediatr.

[25] Ukarapong S, Ganapathy SS, Haidet J, Berkovitz G (2014). Childhood hypophosphatasia with homozygous mutation of ALPL. Endocr Pract.

[26] No E, Zhou Y, Loopstra CA (2000). Sequences upstream and downstream of two xylem-specific pine genes influence their expression. Plant Sci.

[27] Gonzalez F (1999). Early pregnancy diagnosis in goats by determination of pregnancy-associated glycoprotein concentrations in plasma samples. Theriogenology.

[28] Whyte MP (2016). Asfotase Alfa Treatment Improves Survival for Perinatal and Infantile Hypophosphatasia. J Clin Endocrinol Metab.

[29] Weinreb MM, Sharav Y (1964). Tooth Development in Sheep. Am J Vet Res.

[30] Vincent AE (2016). The Spectrum of Mitochondrial Ultrastructural Defects in Mitochondrial Myopathy. Sci Rep.

[31] Scheerlinck JP, Snibson KJ, Bowles VM, Sutton P (2008). Biomedical applications of sheep models: from asthma to vaccines. Trends Biotechnol.

[32] Delmas PD (1990). Biochemical markers of bone turnover for the clinical assessment of metabolic bone disease. Endocrinol Metab Clin North Am.

[33] Pastoureau P, Vergnaud P, Meunier PJ, Delmas PD (1993). Osteopenia and bone-remodeling abnormalities in warfarin-treated lambs. J Bone Miner Res.

[34] Parfitt AM (1984). The cellular basis of bone remodeling: the quantum concept reexamined in light of recent advances in the cell biology of bone. Calcif Tissue Int.

[35] Whyte MP (2016). Asfotase alfa therapy for children with hypophosphatasia. JCI Insight.

[36] Foster BL (2014). Rare bone diseases and their dental, oral, and craniofacial manifestations. J Dent Res.

[37] Koressaar T, Remm M (2007). Enhancements and modifications of primer design program Primer3. Bioinformatics.

[38] Untergasser A (2012). Primer3–new capabilities and interfaces. Nucleic Acids Res.

[39] Zuker M (2003). Mfold web server for nucleic acid folding and hybridization prediction. Nucleic Acids Res.

[40] Shao S (2018). Multiplexed sgRNA Expression Allows Versatile Single Nonrepetitive DNA Labeling and Endogenous Gene Regulation. ACS Synth Biol.

[41] Perrien DS (2007). Inhibin A is an endocrine stimulator of bone mass and strength. Endocrinology.

