# Supplementary material for: Genetic engineering a large animal model of human hypophosphatasia in sheep
Source: Sci Rep. 2018 Nov 16;8:16945. doi: 10.1038/s41598-018-35079-y (PMC6240114; doi:10.1038/s41598-018-35079-y)
Supplement: Supplementary file 1 — Supplementary Information [file 41598_2018_35079_MOESM1_ESM.pdf]

SUPPLEMENTARY INFORMATION FOR:

Genetic engineering a large animal model of human hypophosphatasia in sheep

Authors: Diarra K. Williams[1#], Carlos Pinzón[1#], Shannon Huggins[1], Jane H. Pryor[1], Alyssa Falck[2], Forrest Herman[1], James Oldeschulte[1], Michael B. Chavez[3], Brian L. Foster[3], Sarah H White[4], Mark E. Westhusin[1], Larry J. Suva[1], Charles R. Long[1], and Dana Gaddy*[3]

Affiliations:

[1] Department of Veterinary Physiology and Pharmacology, College of Veterinary Medicine and Biomedical Sciences, Texas A&M University, College Station, TX 77843

[2] Department of Veterinary Integrative Biosciences, College of Veterinary Medicine and Biomedical Sciences, Texas A&M University, College Station, TX 77843

[3]Division of Biosciences, College of Dentistry, The Ohio State University, Columbus, OH, USA.

[4]Department of Animal Science, College of Agriculture and Life Sciences, Texas A&M University, College Station, TX 77843

* Corresponding author. Dr. Dana Gaddy, Department of Veterinary Integrative Biosciences, College of Veterinary Medicine and Biomedical Sciences, Texas A&M University, College Station, TX 77843; 979-862-9134; dgaddy@cvm.tamu.edu

[#] Authors contributed equally to this work.

Supplementary Information

```
Human  1    MISPFLVLAIGTCLTNSLVPEKEKDPKYWRDQAQETLKYALELQKLNTNVAKNVIMFLGDGMGVSTVTAARILKGQLHHN  80
Sheep  1    MISPFLVLAIGTCLASSLVPEKEKDPKYWRDQAQQTLKNALRLQTLNTNVAKNVIMFLGDGMGVSTVTAARILKGQLHHN  80
Mouse  1    MISPFLVLAIGTCLTNSFVPEKERDPSYWRQQAQETLKNALKLQKLNTNVAKNVIMFLGDGMGVSTVTAARILKGQLHHN  80

Human  81   PGEETRLEMDKFPFVALSKTYNTNAQVPDSAGTATAYLCGVKANEGTVGVSAATERSRCNTTQGNEVTSILRWAKDAGKS  160
Sheep  81   PGEETKLEMDKFPYVALSKTYNTNAQVPDSAGTATAYLCGVKANEGTVGVSAATQRSQCNTTQGNEVTSILRWAKDAGKS  160
Mouse  81   TGEETRLEMDKFPFVALSKTYNTNAQVPDSAGTATAYLCGVKANEGTVGVSAATERTRCNTTQGNEVTSILRWAKDAGKS  160

Human  161  VGIVTTTRVNHATPSAAYAHSADRDWYSDNEMPPEALSQGCKDIAYQLMHNIRDIDVIMGGGRKYMYPKNKTDVEYESDE  240
Sheep  161  VGIVTTTRVNHATPSASYAHSADRDWYSDNEMPPEALSQGCKDIAYQLMHNVKDIEVIMGGGRKYMFPKNRTDVEYELDE  240
Mouse  161  VGIVTTTRVNHATPSAAYAHSADRDWYSDNEMPPEALSQGCKDIAYQLMHNIKDIDVIMGGGRKYMYPKNRTDVEYELDE  240

Human  241  KARGTRLDGLDLVDTWKSFKPRHKHSHFIWNRTELLTLDPHNVDYLLGLFEPGDMQYELNRNNVTDPSLSEMVVVAIQIL  320
Sheep  241  KARGTRLDGLNLVDIWKSFKPKHKHSHYVWNRTDLLALDPHTVDYLLGLFEPGDMQYELNRNNVTDPSLSEMVEMAIRIL  320
Mouse  241  KARGTRLDGLDLISIWKSFKPRHKHSHYVWNRTELLALDPSRVDYLLGLFEPGDMQYELNRNNLTDPSLSEMVEVALRIL  320

Human  321  RKNPKGFFLLVEGGRIDHGHHEGKAKQALHEAVEMDRAIGQAGSLTSSEDTLTVVTADHSHVFTFGGYTPRGNSIFGLAP  400
Sheep  321  NKNPKGFFLLVEGGRIDHGHHEGKAKQALHEAVEMDQAIGQAGAMTSVEDTLTVVTADHSHVFTFGGYTPRGNSIFGLAP  400
Mouse  321  TKNLKGFFLLVEGGRIDHGHHEGKAKQALHEAVEMDQAIGKAGAMTSQKDTLTVVTADHSHVFTFGGYTPRGNSIFGLAP  400
                                                 Ile>Met
Human  401  MLSDTDKKPFTAILYGNGPGYKVVGGERENVSMVDYAHNNYQAQSAVPLRHETHGGEDVAVFSKGPMAHLLHGVHEQNYV  480
Sheep  401  MVSDTDKKPFTAILYGNGPGYKVVGGERENVSMVDYAHNNYQA---------------VAVFAKGPMAHLLHGVHEQNYI  465
Mouse  401  MVSDTDKKPFTAILYGNGPGYKVVDGERENVSMVDYAHNNYQAQSAVPLRHETHGGEDVAVFAKGPMAHLLHGVHEQNYI  480

Human  481  PHVMAYAACIGANLGHCAPASSAGSLAA---------------GPLLLALALYPLSVLF-------------  524
Sheep  466  PHVMAYAACIGANRDHCA---SASSPLPargprkpksachppgSPLESSTAARSVAGLQpppppaapfwptg  534
Mouse  481  PHVMAYASCIGANLDHCAWAGSGSAPSP---------------GALLLPLAVLSLRTLF-------------  524
```

Supplemental Fig. 1. Amino Acid sequence alignment of human, sheep and mouse TNSALP protein sequences. Conserved Exon 10 Ile342 > Met for targeted mutation is shown.

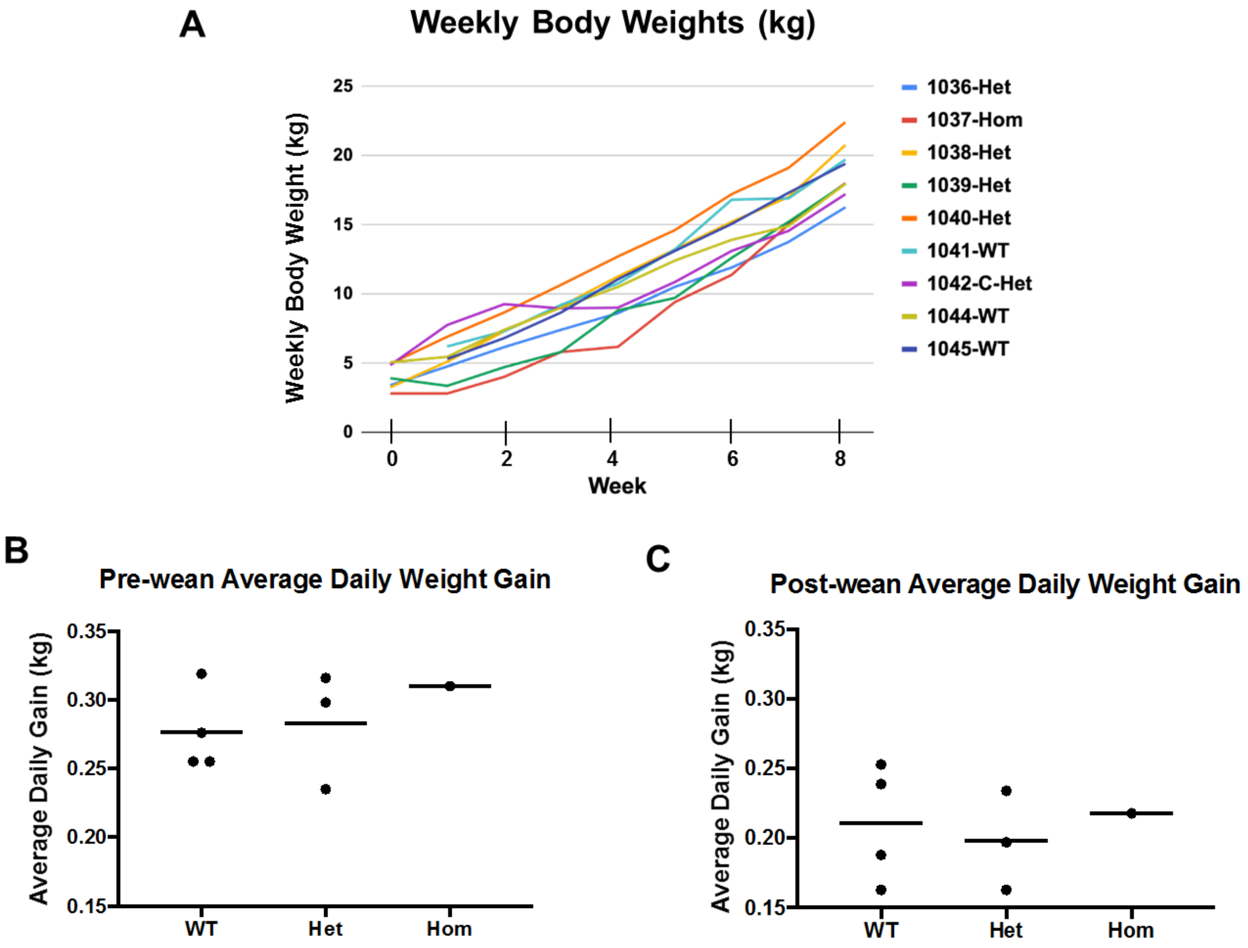

Supplemental Fig. 2 Longitudinal weight gain was similar across all genotypes. Weights were measured at birth and weekly during life. **(A)** Individual preweaning weekly body weights are plotted for all 9 animals, showing similar growth rates over the first 8 weeks of life. Average daily weight gain for each animal is plotted by genotype during **(B)** pre-weaning and **(C)** post-weaning to 6 months of age. No significant difference in weight gain by genotype is observed.

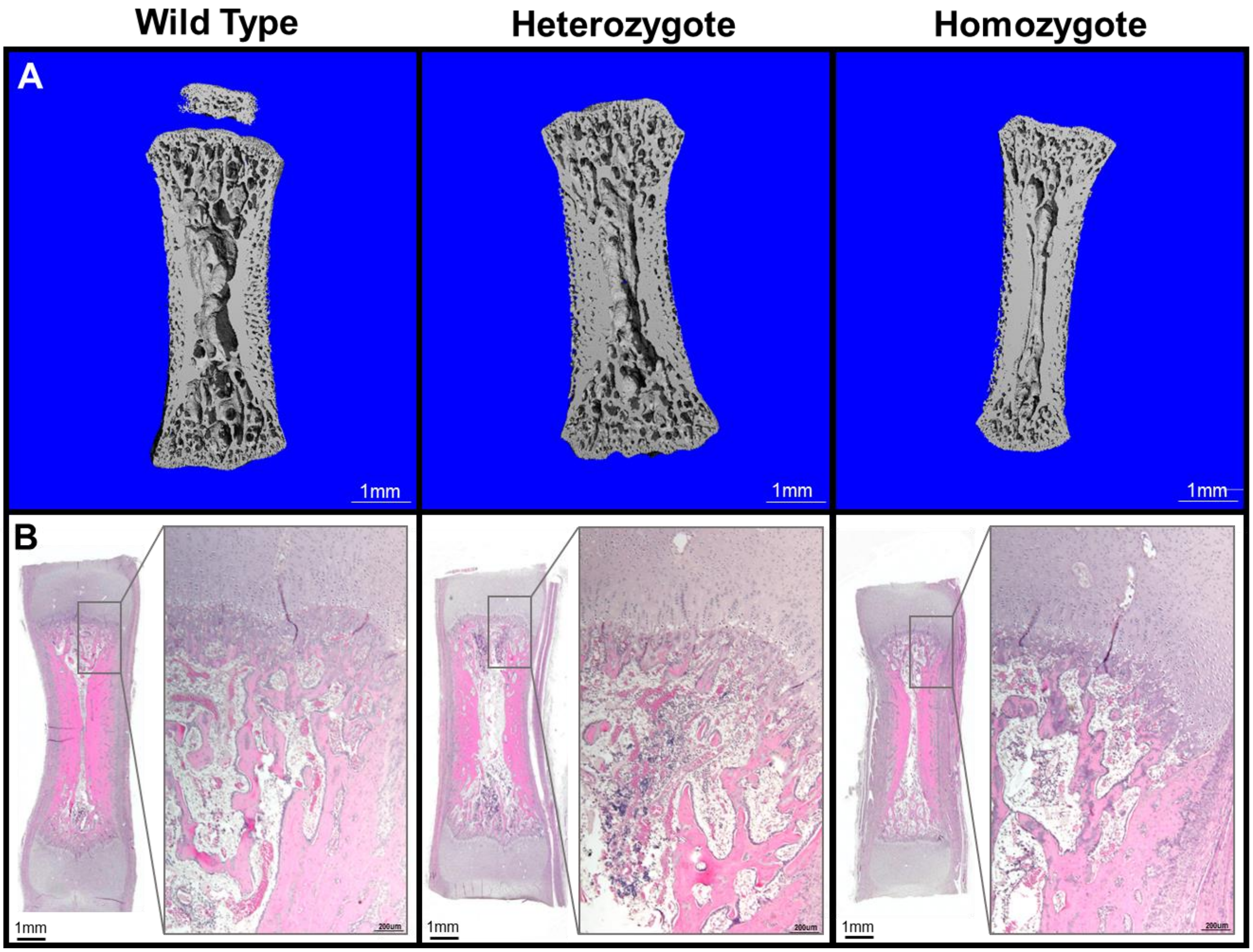

Supplemental Fig. 3 MicroCT reconstructions and histology of *ALPL* c.1077C>G targeted sheep vertebrae (A). 3D renderings of individual caudal vertebrae from wild type, heterozygous and homozygote lambs. Images are sagitally cut to show the extent of mid-shaft cortical and trabecular bone in each vertebral body. (**B**) H&E stained histologic sections from the same wild type, heterozygous and homozygote vertebrae shows a similar sagittal cross-sectional view of the vertebrae representative of each genotype. Insets show developing bone formation, teams of osteoblasts lining trabecular spicules and osteons in the developing sheep cortical bone.

Supplemental Table 1. Minimal potential off-target sites using the ALPL c1077C>G guide #1 sequence.

Sequence	PAM	Score (%)	Gene	Chromosome	Strand	Position	bp Mis-match
GATGGACCAGGCCATCGGGC	AGG	100	ENSOARG00000008507	chr2	-1	244078281	0
GCTGGACCAGGCCATCGGGG	AGG	8.611956522		chr11	-1	33997742	2
GAAGAAGCAAGCCATCGGGC	AAG	0.924515551		chr1	-1	246604745	4
CAGGGACCACGCCATCGGGT	CGG	0.859266382		chr24	-1	41705814	4
CTGGGACCAGGACATCGGGC	AGG	0.796410829		chr11	1	13803103	4
CTGGGACCAGGACATCGGGC	AGG	0.796410829		chr11	1	13798153	4
GTTGGCGGAGGCCATCGGGC	TGG	0.595983173	ENSOARG00000001576	chr26	-1	32197030	4
TAGGGACCCGGCCATCGGGG	CGG	0.56287496		chr3	-1	219963922	4
GCTGGGCGAGCCCATCGGGC	AGG	0.526959664		JH924580.1	1	31944	4
GCTGGGCCCCGCCATCGGGC	CAG	0.525048121	ENSOARG00000018751	chr24	-1	24785660	4
GCTGAGCCAGCCCATCGGGC	AAG	0.522357396		chr11	-1	18680004	4

ENSOARG00000008507= *ALPL* gene
